# siRNA Specificity: RNAi Mechanisms and Strategies to Reduce Off-Target Effects

**DOI:** 10.3389/fpls.2020.526455

**Published:** 2021-01-28

**Authors:** Julia Neumeier, Gunter Meister

**Affiliations:** Regensburg Center for Biochemistry (RCB), Laboratory for RNA Biology, University of Regensburg, Regensburg, Germany

**Keywords:** siRNAs, microRNAs, off-target effects, RISC, RNAi

## Abstract

Short interfering RNAs (siRNAs) are processed from long double-stranded RNA (dsRNA), and a guide strand is selected and incorporated into the RNA-induced silencing complex (RISC). Within RISC, a member of the Argonaute protein family directly binds the guide strand and the siRNA guides RISC to fully complementary sites on-target RNAs, which are then sequence-specifically cleaved by the Argonaute protein—a process commonly referred to as RNA interference (RNAi). In animals, endogenous microRNAs (miRNAs) function similarly but do not lead to direct cleavage of the target RNA but to translational inhibition followed by exonucleolytic decay. This is due to only partial complementarity between the miRNA and the target RNA. SiRNAs, however, can function as miRNAs, and partial complementarity can lead to miRNA-like off-target effects in RNAi applications. Since siRNAs are widely used not only for screening but also for therapeutics as well as crop protection purposes, such miRNA-like off-target effects need to be minimized. Strategies such as RNA modifications or pooling of siRNAs have been developed and are used to reduce off-target effects.

## Introduction

Double-stranded RNA (dsRNA) as trigger for RNA interference (RNAi) has been discovered decades ago in plants and nematodes (Baulcombe, 1996; Fire et al., 1998). Although these organisms are rather distant, the underlying mechanisms are remarkably conserved. dsRNA is generated by transcription or enzymes such as RNA-dependent RNA polymerases (RdRPs), which use single-stranded RNA as a template to generate long dsRNA (Meister and Tuschl, 2004; Mello and Conte, 2004; Sharp and Zamore, 2000). This RNA is further processed to short interfering RNAs (siRNAs), which serve as guides for the RNA-induced silencing complex (RISC) that binds and sequence-specifically cleaves complementary target RNAs (Zamore and Haley, 2005). This process is commonly referred to as RNAi. However, long dsRNA is toxic for animal organisms with more sophisticated immune systems that are capable of sensing long dsRNA as “foreign” as such RNAs could, for example, result from viral infections (Schlee and Hartmann, 2016). However, a breakthrough was reached when it was found that short siRNAs bypass immune sensing and can be used for gene knockdown also in higher organisms such as mammals (Elbashir et al., 2001). Besides broad usage in basic research, siRNAs have now been developed to target genes for therapy and indeed the first siRNAs reached the market (Dorsett and Tuschl, 2004; Sheridan, 2017). In addition to the therapeutic use in mammals, RNAi is also being explored as crop protection agent (Zhang et al., 2017). dsRNA directed against pests such as fungi, nematodes, or insects is sprayed onto the leaves of plants and upon uptake selectively affects growth of distinct target species. Since dsRNA is species specific, is a natural product, and with no genetically modified organism needs to be generated, such strategies are considered highly promising next-generation plant protection agents. Nevertheless, both for human disease and for plant protection purposes, high specificity is critical and off-target effects need to be minimized (Seok et al., 2018). The following chapters will summarize principles of small RNA functions and highlight strategies to reduce off-target effects in gene knockdown experiments.

## RNAi Components in Animals and Plants

Both in animals and plants, long dsRNA is processed to double-stranded siRNAs by Dicer-like enzymes (Bernstein et al., 2001; Grishok et al., 2001; Ketting et al., 2001). In *Arabidopsis thaliana*, four Dicer-like enzymes exist (referred to as DCL1-4), which are specialized for the generation of different classes of small RNAs (Bologna and Voinnet, 2014). DCL1 processes primary microRNAs (miRNA) to 21-nt-long mature miRNAs. DCL2 is involved in antiviral strategies and cleave viral dsRNA to 21/22-nt-long siRNAs, which target viral RNAs. DCL3 functions in silencing processes targeting transposable elements and produces siRNAs of about 24 nt in length. Finally, DCL4 generates 21 nt transacting siRNAs (tasiRNAs), which silence specific endogenous genes. Except for DCL1 that engages already-folded and dsRNA precursors, DCL2-4 cooperate with specialized RdRPs that generate the dsRNA substrates from single-stranded transcripts [for more information on plant Dicer enzymes, see Bologna and Voinnet (2014), Fukudome and Fukuhara (2017)].

**FIGURE 1:**
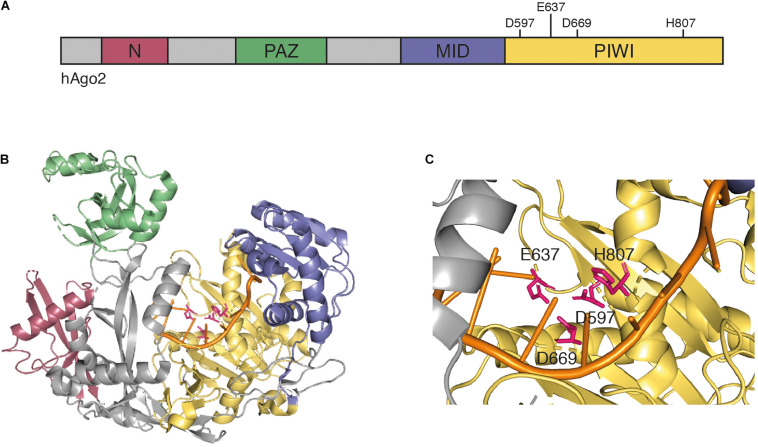
**(A)** Schematic representation of human Ago2. Argonaute proteins contain four conserved domains: the N domain (red), the PAZ domain (green), the MID domain (blue), and the PIWI domain (yellow). The PIWI domain contains the four catalytic residues D597, E637, D669, and H807 that are required for mRNA target cleavage. **(B)** Crystal structure of human Ago2 loaded with a siRNA (PDB ID 5JS1) (Schirle et al., 2016). The four domains are colored as in panel **(A)**, the catalytic residues are highlighted in pink, and the 5′ region of the loaded siRNA is shown in orange. **(C)** Detailed view of catalytic center of the Ago2 structure from panel **(B)**.

In animals, Dicer enzymes are typically less diverse (Meister and Tuschl, 2004). Dicer enzymes belong to the RNase III enzyme family, which recognizes the ends of long dsRNAs and cleave the RNA about 21 nt from the end (Treiber et al., 2019). Dicer-like enzymes possess two catalytic RNase III domains, which cleave both strands and due to their positioning on the dsRNA, leave two nucleotides 3′ overhangs (Filipowicz, 2005; Treiber et al., 2019). In subsequent steps, commonly referred to as RISC loading, a member of the Argonaute protein family recognizes particularly the 3′ overhangs and selects one strand of the duplex to become the guide strand (also referred to as the antisense strand). The other strand, referred to as passenger strand, is degraded (Dueck and Meister, 2014; Ipsaro and Joshua-Tor, 2015; Sheu-Gruttadauria and MacRae, 2017). While structural information for most Ago proteins is lacking (including all plant Ago proteins), human Ago2 is reasonably well understood and is presented as an example for general structural features of Ago proteins. Argonaute proteins are structurally highly conserved and typically contain four domains (Figures 1A,B), as follows: the N domain, which has been implicated in siRNA duplex unwinding (Kwak and Tomari, 2012), the PAZ domain that anchors the 3′ end of the selected guide strand (Lingel et al., 2003; Ma et al., 2004; Yan et al., 2003), the MID domain that binds the 5′ end of the guide strand (Ma et al., 2005; Parker et al., 2004), and the PIWI domain. The PIWI domain has structural similarities to RNase H, which cleaves RNA molecules in RNA-DNA hybrids (Song et al., 2004; Wang et al., 2009; Yuan et al., 2005). Thus, some, but not all Argonaute proteins are endonucleases that cleave the target RNA in siRNA-target RNA hybrids using a catalytic tetrad in their active centers (Nakanishi et al., 2012) (Figure 1C). These proteins are referred to as Slicer enzymes (Liu et al., 2004; Meister et al., 2004). Furthermore, structural work revealed that MID domains of animal and plant Ago proteins display a sequence bias regarding the 5′ terminal nucleotide, which is important for sorting specific classes of small RNAs into their correct silencing pathways (Frank et al., 2010; Frank et al., 2012; Mi et al., 2008).

Plant Argonaute proteins are functionally diverse and are involved in various different gene silencing processes (Carbonell, 2017). In *A. thaliana*, Ago1, Ago7, and Ago10 bind to miRNAs and silence target genes. Ago4 has been implicated in RNA-directed DNA methylation and chromatin is epigenetically modified by this pathway. Generally, through their small RNA partner, plant Ago proteins are also involved in antiviral or bacterial defense mechanisms as well as responses to herbivore attack (Pradhan et al., 2017; Sibisi and Venter, 2020).

In plants as well as some animal species, which tolerate long dsRNA, the RNAi signal can be amplified by RdRPs (Maida et al., 2011). These enzymes use a siRNA strand bound to its target RNA as primer and synthesize the complementary strand to the target RNA resulting in a long dsRNA, which again enters Dicer processing and a second wave of siRNAs against a specific target is generated. Furthermore, in plants and also in some animal species, particularly nematodes such as *Caenorhabditis elegans*, siRNAs can be actively transported between cells and tissues [see, for example, (Das et al., 2019) for more information on this broad topic]. Remarkably, siRNA signals can also be inherited and target RNAs can be silenced over many generations. This process has been studied in *C. elegans* and is known as transgenerational gene silencing (TGS) [for more information on this exciting topic, please see, for example, (Iwasaki et al., 2015; Lev et al., 2019; Schraivogel and Meister, 2014)].

## miRNA-Guided Gene Silencing

MiRNAs are found in almost all plants and animals and in contrast to siRNAs, are transcribed from distinct miRNA genes (Bartel, 2009; Kim et al., 2009). RNA polymerase II transcription results in capped and poly-adenylated primary miRNA transcripts (pri-miRNAs), which are recognized and processed by the nuclear microprocessor containing the RNase III enzyme Drosha and its interaction partner DGCR8 (Cai et al., 2004; Denli et al., 2004; Gregory et al., 2004; Han et al., 2004; Landthaler et al., 2004; Lee et al., 2003; Lee et al., 2004). Drosha/DGCR8 do not exist in plants and therefore DCL1 processes primary miRNA hairpins to mature miRNAs already in the nucleus of plant cells (Bologna and Voinnet, 2014; Bologna et al., 2018; Fukudome and Fukuhara, 2017).

**FIGURE 2:**
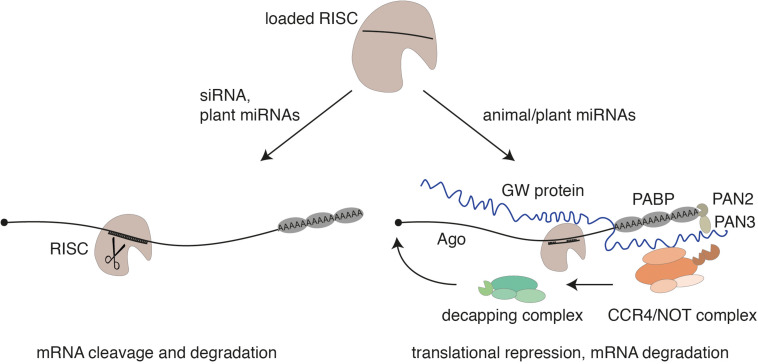
The guide strand of the siRNA or a miRNA is loaded into an Argonaute protein. In case of a perfect complementarity through siRNAs or miRNAs (which is often observed in plants), a catalytically active Argonaute protein cleaves the mRNA as part of RISC (left). As miRNAs in animals are only partially complementary to their target RNAs, Slicer-facilitated cleavage is impaired. In this case, Argonaute recruits a member of the GW protein family. These proteins mediate the interaction with further downstream acting factors like poly-(A)-binding proteins (PABPs) or the deadenylase complexes PAN2/PAN3 and CCR4/NOT. This leads to translational repression, deadenylation, decapping, and 5′–3′ exonucleolytic decay of the mRNA. Translational repression by miRNAs has also been observed for plant miRNAs.

Within pri-miRNAs, the miRNA strand itself is embedded in the stem of a local hairpin and the microprocessor cleaves the hairpin at the base of the stem. The resulting hairpin structure, referred to as miRNA precursor (pre-miRNA), is exported to the cytoplasm by the export receptor Exportin-5 in animals (Bohnsack et al., 2004; Lund et al., 2004; Yi et al., 2003). In the cytoplasm, Dicer binds to the end of the hairpin and cleaves off an approx. 21-nt-long dsmiRNA intermediate, which is reminiscent of a siRNA duplex described above (Zhang et al., 2002). Consistently, RISC loading processes are similar to siRNAs and both in plants and in animals require the action of heat shock protein 90 (HSP90) that holds Ago proteins in a loading competent open conformation (Dueck and Meister, 2014; Iki et al., 2012; Iwasaki et al., 2010; Miyoshi et al., 2010). Moreover, miRNAs function like siRNAs in case the miRNA and the target RNA are fully or almost fully complementary (Doench et al., 2003). This mechanism is predominant in plants (Song et al., 2019). In animals, however, target RNA binding as well as the mechanism of gene silencing is different. MiRNA-target sites are typically located in the 3′ untranslated region (UTR) of mRNAs (Bartel, 2009). Nucleotides 2–8 of the miRNA represent the seed sequence, which is generally fully complementary to the target site while the remaining sequence is often only partially paired (Rajewsky and Socci, 2004). This incomplete pairing prevents Slicer-mediated cleavage as it is observed also in siRNA-guided knockdown studies. Instead, Argonaute proteins recruit a member of the GW protein family, which coordinates the following steps in miRNA-guided gene silencing (Behm-Ansmant et al., 2006; Jakymiw et al., 2005; Liu et al., 2005; Meister et al., 2005). GW proteins are characterized by glycine-tryptophane repeats and are referred to as TNRC6 proteins in mammals (Pfaff and Meister, 2013; Pfaff et al., 2013). GW proteins establish interactions with the poly(A) tail of the mRNAs as well as deadenylase complexes including the CCR4/NOT complex or PAN2/3 leading to translational repression, deadenylation of the mRNA, and, finally, to the removal of the 5′ cap by decapping enzymes (Figure 2). The unprotected mRNA is then degraded by 5′–3′ exoribonucleases [for more details, see Braun et al. (2013), Jonas and Izaurralde (2015), Krol et al. (2010)]. Translation repression without site-specific cleavage has also been observed in plants of target sites that are fully complementary but located in the 3′ UTR of mRNAs (Brodersen et al., 2008). Since GW proteins are not conserved in plants, the extent of this type of miRNA action remains to be further investigated in plants (Song et al., 2019).

## miRNA-Like Off-Target Effects in RNAi Strategies

Although miRNA-guided gene silencing is distinct from siRNA-guided knockdown experiments, the pathways are intertwined and miRNAs can function as siRNAs and vice versa. This is particularly important for off-target effects observed in RNAi experiments (Seok et al., 2018). SiRNAs may, in addition to their fully complementary on-targets, bind to an undefined number of miRNA-like target sites in 3′ UTRs of mRNAs using their seed sequence. This will lead to silencing and unwanted off-target effects. Since such sequences are only 6–7 nt long, these unspecific target sites are hardly predictable and are thus very difficult to avoid. Indeed, miRNA-like off-target effects are highly problematic in large-scale RNAi screening approaches, and many hits are false positive and caused by off-target effects [e.g., (Birmingham et al., 2006; Buehler et al., 2012; Fedorov et al., 2006; Jackson et al., 2006b)]. Thus, strategies that control for or even reduce or eliminate such off-target effects are urgently needed.

In RNAi-mediated pest control, such off-target effects might not be predominantly problematic for the plant system since such a translational control system might be rather rare. However, in strategies, in which plants express si- or shRNAs that are taken up by animals and are toxic to defined species, off-target effects need to be considered. For example, non-target animals might incorporate these RNAs as well and, although perfect complementary target RNAs are absent, the expression of partially complementary sites could be affected through the endogenous miRNA system.

## Strategies to Reduce miRNA-Like Off-Target Effects

SiRNAs are typically designed to avoid complementary sequences to other RNAs besides the on-target. However, miRNA-like seed matches are difficult to predict because they statistically occur very frequently on mRNAs and not all such matches are always leading to significant knockdown effects. A conclusive strategy to monitor such effects are whole transcriptome sequencing in case target organisms and cells are identifiable. However, molecules with strongly reduced off-target effects would be the most desirable approach. To reduce miRNA-like off-target activities, two main strategies have been developed (Jackson and Linsley, 2010; Seok et al., 2018). First, siRNA guide strands are chemically modified within their seed region particularly at position 2 from the 5′ end (Jackson et al., 2006a). Such modifications are either 2′-*O*-methylations or locked nucleic acid incorporations, in which the 2′-OH is chemically linked to the 4′ carbon of the ribose (Elmen et al., 2005). Both modifications weaken the interaction between the guide strand and the target. Since seed matches are short, such interactions are much stronger affected by this mild destabilization than siRNAs, which are typically fully complementary to their on-target. Thus, miRNA-like off-target interactions are reduced while on-target silencing is not compromised. In addition to the modification at position 2, other modifications have also been explored [for more details, please see Seok et al. (2018)]. A second approach to reduce off-target effects is pooling of multiple siRNAs. It is important to notice that miRNA-like off-target effects are specific to individual sequences. Thus, reducing the concentration of the applied siRNAs will also reduce miRNA-like off-target effects. This could be achieved by administration of very low concentrations (Persengiev et al., 2004). However, this would also directly affect on-target activity. An elegant way of lowering concentrations of siRNAs is siRNA pooling. Individual siRNAs within such a pool are directed against the same on-target at different positions, but each individual siRNA has a unique off-target signature. Consequently, all siRNAs act synergistically on the same on-target RNA. In complex pools, concentrations of individual siRNAs are very low and thus miRNA-like off-target effects are diluted out and cannot be measured anymore. Based on these ideas, three main pooling strategies are currently used. First, in the so-called smartPools, four individual siRNAs are combined. However, the complexity of such pools is low and thus the desired dilution effects are often not very pronounced. In contrast, endoribonuclease-produced siRNA pools (esiRNAs) are generated *in vitro* by recombinant RNase III digestion of long dsRNA (Kittler et al., 2005). These pools are then applied to cell cultures and, since these highly complex pools contain hundreds of different siRNAs, sequence-specific off-targets are not observed (Hannus et al., 2014; Kittler et al., 2005). A third strategy are so-called siPOOLs, which are highly complex but in contrast to esiRNAs, well defined. Up to 30 different siRNAs are designed and generated *in vitro* and such pools eliminate off-target effects even when a single siRNA with a pronounced off-target is included into the pool (Hannus et al., 2014).

Chemical modifications are the preferred choice when siRNAs are used for therapeutic purposes. For drug development, single and well-defined molecule species are preferred since broad toxicological validations are required during clinical trials and final approval. SiRNA pooling strategies are preferred in individual knockdown studies for research purposes or in genome-wide RNAi screening studies. Such pools are cost-efficient and thus genome-scale libraries are available.

## Conclusion for RNA-Based Crop Protection and Outlook

Plants and animals with rather primitive immune systems tolerate long dsRNA and process it to siRNAs for gene silencing. One strategy in RNA-based crop protection is to spray dsRNA directed against pest-specific genes onto plants (Cai et al., 2018). Fungi or herbivores will take up these RNAs and process them to complex siRNA mixtures similar to esiRNA pools. This will kill or affect growth of the pathogens. Since such complex pools are naturally generated from dsRNAs in nematodes, insects, or fungi, miRNA-like off-target activity might be neglectable, when dsRNA is applied. In higher organisms such as mammals, the dsRNA will be fully degraded while transitioning through the digestive tract and only free nucleosides will be taken up. Thus, the administration of dsRNA to plants is an elegant and presumably very safe way of plant protection. SiRNAs are designed sequence specifically, and effects on other even highly related species could be minimized. Furthermore, since dsRNA is a natural product that is present in human diet, it might be better accepted by local communities than other plant protection strategies including the generation of genetically modified organisms (GMOs) or the use of conventional pesticides.

## Author Contributions

GM structured and wrote the text. JN wrote the text and designed figures. Both authors contributed to the article and approved the submitted version.

## Conflict of Interest

GM is a co-founder of siTOOLs biotech. The remaining author declares that the research was conducted in the absence of any commercial or financial relationships that could be construed as a potential conflict of interest.
